# Extracellular Vesicles from *Leishmania*-Infected Macrophages Confer an Anti-infection Cytokine-Production Profile to Naïve Macrophages

**DOI:** 10.1371/journal.pntd.0003161

**Published:** 2014-09-18

**Authors:** André Cronemberger-Andrade, Luciana Aragão-França, Cintia Figueiredo de Araujo, Viviane Junqueira Rocha, Mariana da Cruz Borges-Silva, Cláudio P. Figueiras, Pablo R. Oliveira, Luiz A. R. de Freitas, Patrícia S. T. Veras, Lain Pontes-de-Carvalho

**Affiliations:** Centro de Pesquisas Gonçalo Moniz, Fundação Oswaldo Cruz, Salvador, Brazil; The Ohio State University, United States of America

## Abstract

**Background:**

Extracellular vesicles (EVs) are structures with phospholipid bilayer membranes and 100–1000 nm diameters. These vesicles are released from cells upon activation of surface receptors and/or apoptosis. The production of EVs by dendritic cells, mast cells, macrophages, and B and T lymphocytes has been extensively reported in the literature. EVs may express MHC class II and other membrane surface molecules and carry antigens. The aim of this study was to investigate the role of EVs from *Leishmania*-infected macrophages as immune modulatory particles.

**Methodology/Principal Findings:**

In this work it was shown that BALB/c mouse bone marrow-derived macrophages, either infected *in vitro* with *Leishmania amazonensis* or left uninfected, release comparable amounts of 50–300 nm-diameter extracellular vesicles (EVs). The EVs were characterized by flow cytometry and electron microscopy. The incubation of naïve macrophages with these EVs for 48 hours led to a statistically significant increase in the production of the cytokines IL-12, IL-1β, and TNF-α.

**Conclusions/Significance:**

EVs derived from macrophages infected with *L. amazonensis* induce other macrophages, which *in vivo* could be bystander cells, to produce the proinflammatory cytokines IL-12, IL-1β and TNF-α. This could contribute both to modulate the immune system in favor of a Th1 immune response and to the elimination of the *Leishmania*, leading, therefore, to the control the infection.

## Author Summary

Leishmaniases are a group of diseases—each one individually called leishmaniasis—that are caused by the protozoan *Leishmania*. They affect millions of people and thousands of dogs in tropical and mediterranean countries. Macrophages are the main cellular hosts of *Leishmania* in the mammalian host, where it is an obligatorily intracellular parasite. In this work, it is shown that mouse bone marrow-derived macrophages, when infected *in vitro* with *Leishmania*, release small (no larger than 300 nm) extracellular vesicles (EVs), in the same way as uninfected macrophages. The EVs from the infected macrophages, however, induce in other macrophages the production of some cell hormones, named cytokines, which are involved with protection of the macrophage against infection and with the development of a protective immune response against the parasite.

## Introduction

Leishmaniases are a disease complex caused by about 21 trypanosomatid protozoa of the genus *Leishmania* [1]–[3]. Parasites of that genus have been co-evolving with their mammals and insect hosts for thousands of years. It is not surprising, therefore, that they are well adapted to these hosts and vectors, persisting and replicating in their tissues, subverting the immune response of the vertebrate host, and spreading to other hosts of the same or of another species. In the mammalian host, the *Leishmania* survives and replicates as amastigotes, mainly inside macrophages [4].

The immunity to *Leishmania* is associated with a parasite-specific Th1 immune response [5], [6]. The release of IL-12 by macrophages and dendritic cells leads to production of IFN-γ by NK cells and differentiation of Th0 cells into Th1 cells, which also release IFN-γ. IFN-γ provides a key stimulus for the development of macrophage resistance to most intracellular pathogens, including the *Leishmania* [7], [8].

Communication between cells occurs by different mechanisms. It can be, for instance, through growth factors, cytokines, nucleotides, lipids, nitric oxide, peptides, and adhesion molecules. An additional mechanism is by means of extracellular vesicles (EVs). The immune system comprises a group of often physically isolated cells that intensely communicate among themselves. The production of EVs by dendritic cells, mast cells, macrophages, and B and T lymphocytes has been extensively described in the literature [9]. These EVs carry the phenotypic characteristics of the cells they originate from. Thus, depending on their origin, they may express MHC class II molecules and may transport antigens [10], [11].

Macrophages infected by microorganisms produce EVs, which can induce inflammatory responses *in vitro* and *in vivo* [12]–[15]. Because of their immune modulatory activities, EVs are being investigated as components of future vaccines [16]–[19]. Three main types of EVs have been described in the literature: exosomes, microparticles and apoptotic bodies [10].

Exosomes are small vesicles of endosomal origin that are released by various cell types, including macrophages, and that are capable of modulating the immune response [20]. They range in size from 40 to 100 nm in diameter and have rounded and flattened morphology [21]. When derived from antigen-presenting cells, they can promote the adaptive immune response by presenting MHC class II molecule-peptide complexes to naïve CD4^+^ T cells, as well as by inducing specific CD8^+^ T-cell responses [22]–[25].

It has been demonstrated that exosomes may carry antigens from tumour cells [26] and from cells infected with intracellular microorganisms such as mycobacteria [27], cytomegalovirus [28], and *Leishmania* [29].

Microparticles are spherical bodies formed from the budding off of cell plasma membranes after cell activation and apoptosis, with diameters ranging from 100 to a 1000 nm, and which contain cytoplasmic components, such as proteins and nucleic acids [30]. They can be characterized by the presence of surface molecules from the cells from which they were derived [10], [31]. Microparticles from macrophages infected with *Mycobacterium tuberculosis* carry mycobacterium antigens and promote inflammation by activating CD4^+^ T cells [32]. Those from macrophages infected with *Listeria monocytogenes* also carry microorganism antigens and elicit a protective immune response [33]. Some studies suggest that macrophages are activated by microparticles via the toll-like receptor 4 (TLR4) [34].

Unlike other EVs, which are released either by cell activation or in the early stages of apoptosis, apoptotic bodies are formed in the final stages of the apoptotic process. They have larger diameters (1–5 µm) than the other EVs and display phosphatidylserine molecules on their membranes [35]. They have also been shown to induce immune responses [36].

In the present work, EVs obtained from *Leishmania amazonensis*-infected macrophages from BALB/c mice were compared with EVs from uninfected macrophages in terms of eliciting the production of cytokines by naïve macrophages. The BALB/c mouse-*L. amazonensis* combination was chosen because that *Leishmania* species causes a progressive cutaneous disease in that strain of mice, and we hypothesized that macrophage-derived EVs could have infection promoting properties.

It was found, on the contrary, that EVs from *L. amazonensis*-infected macrophages promote the release by other macrophages of cytokines that usually induce inflammatory responses.

## Methods

### Ethics Statement, Animals and Parasites

Four to 8 week-old BALB/c mice were used for collecting resident macrophages and bone marrow cells. The animals received balanced feed and water *ad libitum*. All procedures were performed in accordance with the ethics principles in animal research of the Brazilian law 11784/2008 and were approved by the Ethics Committee for Animal Research of the Gonçalo Moniz Research Centre, Oswaldo Cruz Foundation, Salvador, Brazil (protocol no. 003/2013). *L. amazonensis* (MHOM/BR88/BA/125) parasites were maintained by regular passages in Golden hamsters. The promastigotes derived from tissue amastigotes were grown under sterile conditions at 24°C in Schneider's medium (Schneider's drosophila medium, Sigma-Aldrich Chemical Co., St. Louis, MO, USA) containing 50 µg.mL^−1^ gentamicin and 10% heat-inactivated fetal bovine serum (FBS; Gibco, Grand Island, NY, USA).

### Infection of Macrophages and Preparation of Extracellular Vesicles

Bone marrow cells were differentiated into macrophages by 7–8 days incubation with RPMI (supplemented with 10% FBS, 50 µg/mL of gentamicin, 3.6 g/L of sodium bicarbonate, 25 mM HEPES, 2 mM glutamine and 30% supernatant of culture of GM-CSF–expressing cells), as described in the literature [37]. Two×10^5^ macrophages were incubated with stationary-phase *L. amazonensis* promastigotes at parasite to macrophage ratio of 50∶1 in 1 mL volumes of RPMI supplemented with 10% FBS, 50 µg/mL gentamicin, 3.6 g/L sodium bicarbonate, 25 mM HEPES, 2 mM glutamine (supplemented RPMI) during 6 hours, followed by a washing to remove non-internalized parasites. To isolate the EVs, the infected and control non-infected macrophages were cultured for 9 days in supplemented RPMI. After the 9-day incubation, the culture supernatants were centrifuged at 500 g for 10 minutes at 4°C, 1500 g for 10 minutes at 4°C and 8,000 g for 5 minutes at 4°C to remove residual cells and cellular debris. The EVs present in the supernatants were washed once with Hanks' balanced salt solution (HBSS) by centrifugation at 100,000 g for 45 minutes at 4°C and resuspended in cold HBSS. The protein concentrations in the extracellular vesicle preparations were determined with the BCA kit (Thermo Scientific, Rockford, USA) in accordance with the manufacturer's instructions.

The mean yields of EVmedium and EVLa obtained from 1-mL cultures of 2×10^5^ macrophages, plus or less one standard deviation, were 5.2×10^7^±0.4×10^7^ and 5.0×10^7^×1.2×10^7^ EVs, respectively. These numbers were determined by flow cytometry as described below.

Cells were also cultured over glass coverslips and stained with hematoxylin and eosin (H&E) for determining the infection rate.

The FBS used in the cultures was previously ultracentrifuged at 100.000 g for 4 hours in order to precipitate the EVs it probably had in suspension.

### Characterization of EVs and Bone Marrow-Derived Macrophages, and Enumeration of EVs, by Flow Cytometry

For characterization of the EVs, the macrophages (in cultures inside wells of 24-wells culture plates) were washed twice with 0.15 M phosphate-buffered saline, pH 7.2 (PBS) and incubated with a 2 µM PKH26 (Sigma-Aldrich, St. Louis, MO, USA) solution or with a 20 µM carboxyfluorescein diacetate (CFDA; Sigma-Aldrich, St. Louis, MO, USA) solution in PBS for 2–5 minutes at room temperature with gently shaking. The staining reaction was stopped by adding an equal volume of FBS and incubating for 1 minute at room temperature. The macrophages were then washed once with PBS to remove the staining solution and cultured with supplemented RPMI. The culture supernatants were centrifuged for isolation of the EVs as described above, and the EVs were mixed together with 1 µm-diameter fluorescent beads (Invitrogen, Carlsbad, CA, USA) and analyzed in a flow cytometer.

Monoclonal antibody (mAb)-fluorochrome conjugates (clone M1/70 phycoerythrin—anti-CD11b mAb, clone BM8 phycoerythrin-cyanine—anti-F4/80 mAb, and clone NIMR-4 FITC—anti-mouse MHC II mAb) were from eBioscience (San Diego, CA, USA). Annexin V-FITC and propidium iodide were from Sigma (Apoptosis Detection Kit; Sigma-Aldrich, St. Louis, MO, USA). The flow cytometry technique was carried out as described in the literature [38]. Briefly, the cells or EVs were washed once with flow cytometry buffer (PBS containing 5% FBS), incubated with the conjugates on ice for 20 min and washed twice with flow cytometry buffer. The cell or extracellular vesicle suspensions were then washed once with PBS and analysed in a flow cytometer.

The enumeration of EVs was done by flow cytometry, based on the number of events occurring in a gate for the right-size particles in the time that 500 fluorescent beads (Invitrogen, Carlsbad, CA, USA) were counted. This was performed in four extracellular vesicle preparations from *L. amazonensis*-infected macrophages and four control preparations from non-infected macrophages.

### Treatment of Macrophages with Membrane Vesicles

Peritoneal cells without stimulation with thioglycollate were obtained by washing the peritoneal cavity with 0.9% NaCl containing 20 IU.mL heparin. The cells were centrifuged at 300 g for 10 minutes at 4°C and resuspended in supplemented medium. One million large mononuclear peritoneal cells were distributed in each well of 24-well plates. After a 24-hour incubation period at 37°C, the non-adherent cells were discarded by replacement of the culture supernatants by new medium. More than 90% of the adherent cells were shown to be positive for CD11b and F4/80 by the flow cytometry technique described above (data not shown), a fact that characterized them as being constituted mostly by macrophages. These peritoneal macrophages were incubated for 48 hours with EVs from uninfected bone marrow-derived macrophages (EVmedium) and with EVs from *L. amazonensis*-infected bone marrow-derived macrophages (EVLa) with or without 1 ng/mL bacterial lipopolysacharide (LPS; Escherichia coli 0127:B8, Sigma-Aldridge, St. Louis, MO, USA), at 37°C and 5% CO_2_. In cultures that received EVmedium and EVLa in the absence of LPS, 10 µg/mL of polymyxin B (Sigma-Aldridge, St. Louis, CA, USA) were added to rule out the possibility that a putative LPS contamination could compromise the interpretation of results.

The extracellular vesicle concentrations used in the treatment were the same concentrations in which the EVs were in the macrophage culture supernatants from which they were isolated. The mean numbers of EVmedium and EVLa added to the cultures, plus or less one standard deviation, were 5.2×10^7^±0.4×10^7^ and 5.0×10^7^±1.2×10^7^, respectively. Approximately 52 EV medium and 50 EVLa were, therefore, added per macrophage. The total amounts of protein contained in the added preparations were 11.4±1.1 µg for EV medium and 10.0±1.0 µg for EVLa.

### Macrophage Viability Analysis

The supernatants of cultures in 24-well plates of bone marrow-derived macrophages were replaced by RPMI containing 10% Alamar blue (Invitrogen, Carlsbad, CA, USA) on the 1st, 3rd, 6th, 9th and 12th days after infection with *L. amazonensis*, or to cultures of control uninfected macrophages, in triplicates. After incubation for 4 hours at 37°C and 5% CO_2_, 200 µL volumes of the supernatants were transferred to wells of 96-well microtiter plates and the absorbance for light with wavelengths of 570 and 600 nm was read. As assay controls, wells of 96-well microtiter plates received only culture medium or only medium with Alamar blue.

### Cytokine Detection

The concentrations of cytokines (IL-6, TNF-α, IL-12p70, IL-1β and IL-10) in culture supernatants were determined with the Ready-Set-Go kit (eBioscienses, San Diego, CA, USA), in accordance with the manufacturer's instructions.

### Electron Microscopy

EVs were fixed with sodium cacodylate buffer and 2% paraformaldehyde for 2 hours at room temperature. After fixation, the EVs were centrifuged at 100,000 g for 45 minutes, resuspended in PBS, and passed through filters with 0.22-µm diameter pores. The EVs were adsorbed to copper (Formvar) grids for 20 minutes. Two % phosphotungstenic acid was used for negative staining. The EVs were visualized in a transmission electron microscope (Zeiss EM 109; Munich, Germany).

Macrophages were fixed with 2.5% glutaraldehyde and 2% paraformaldehyde in 0.1 M sodium cacodylate buffer. Post-fixation was performed with 1% osmium tetroxide, 0.8% potassium ferricyanide and 5 mM calcium chloride in 0.1 M sodium cacodylate buffer. Dehydration was performed with acetone and the material mounted in resin (Polybed 812). The resin block was cut in an ultramicrotome, contrasted, and the material observed in the transmission electron microscope.

### Statistical Analyses

To compare the levels of a given cytokine in cultures treated with either EVLa or EVmedium, the following assumptions were made:

1. the distribution of the data was non-gaussian [this assumption was made because the number of different EVLa and EVmedium preparations tested (n = 6) was too small to allow the determination of the type of distribution];
2. the EVLa would stimulate the production of either proinflammatory cytokines or regulatory cytokines, or of both, more than the EVmedium, and the EVs would stimulate the production of one or more of these cytokines so that their concentrations in the culture supernatants would be higher than in the supernatants of unstimulated macrophage cultures.

These two assumptions determined that a directional non-parametric method should be applied. The Wilcoxon's signed-rank test was therefore used to determine the statistical significance of differences in cytokine levels in six independent experiments, and the Mann-Whitney's U test was used to determine the statistical significance of differences in macrophage viability and extracellular vesicle numbers. The differences were considered significant when *P* was ≤0.05.

## Results

### Characterization of Macrophages of Myeloid Origin and of Their Infection by *L. amazonensis*

**Figure 1:**
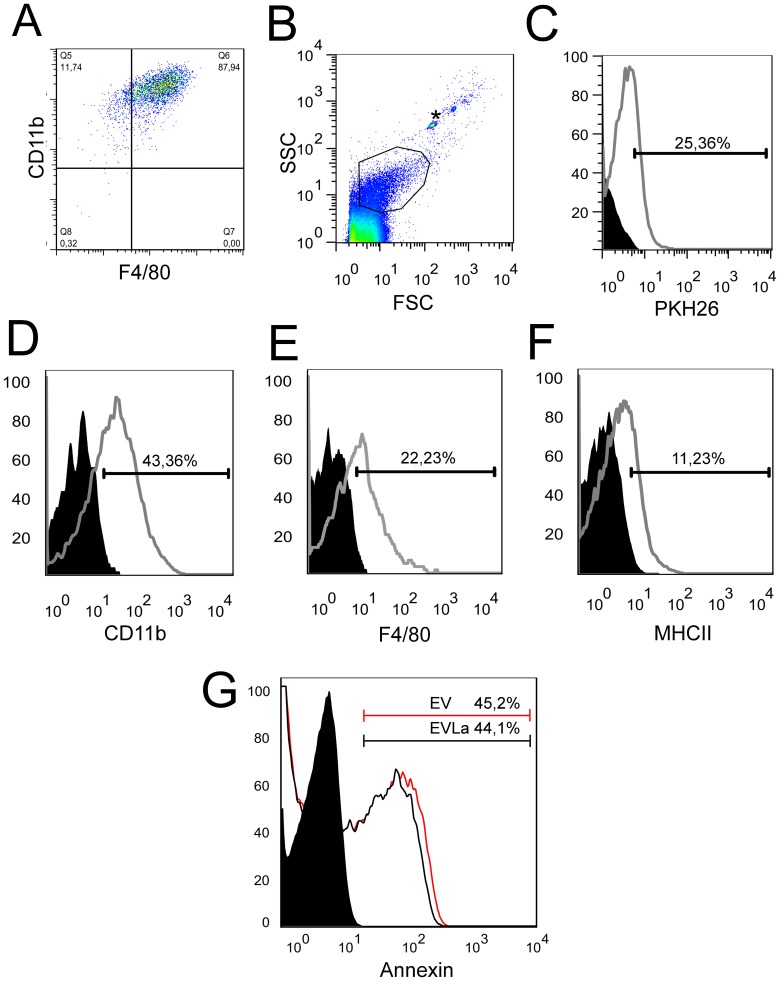
Flow cytometry analysis of bone marrow-derived macrophages and their extracellular vesicles (EVs). A, macrophages obtained by differentiation from bone marrow cells treated with GM-CSF were phenotyped with anti-F4/80 and anti-CD11b. 87.9% of the cells were double labeled. B, side scatter (SSC) and forward scatter (FSC) analysis showing the selection of the EV population, which is demarcated in the highlighted area. Beads with 1 µm in diameter (whose position is indicated by the asterisk) were used as reference. C, EVs derived from cell membranes labelled with PKH26. Curves with areas underneath in black were produced by unlabelled EVs and with areas underneath in white were produced by labelled EVs. D, E, and F, EVs were labelled with anti-CD11b, anti-F4/80 and anti-MHC type II, respectively. The curves with areas underneath in black were produced by unlabelled EVs and curves with areas underneath in white were produced by labelled EVs. G, annexin V-labelled EVs that had been collected on the 9th day of macrophage culture. The curves with areas underneath in black were produced by unlabelled EVs from uninfected macrophages and from infected macrophages, curves with areas in gray were produced by labelled EVs from uninfected macrophages, and curves with areas in red were produced by labelled EVs from infected macrophages.

The cells differentiated from bone marrow cells were stained with surface antibodies specific for murine monocytes (anti-CD11b and anti-F4/80) for the purpose of evaluating their phenotype by flow cytometry. A percentage of 88% of cells double–labelled for anti-F4/80 and anti-CD11b was observed (Fig. 1A), indicating that at least the vast majority of them were macrophages.

**Figure 2:**
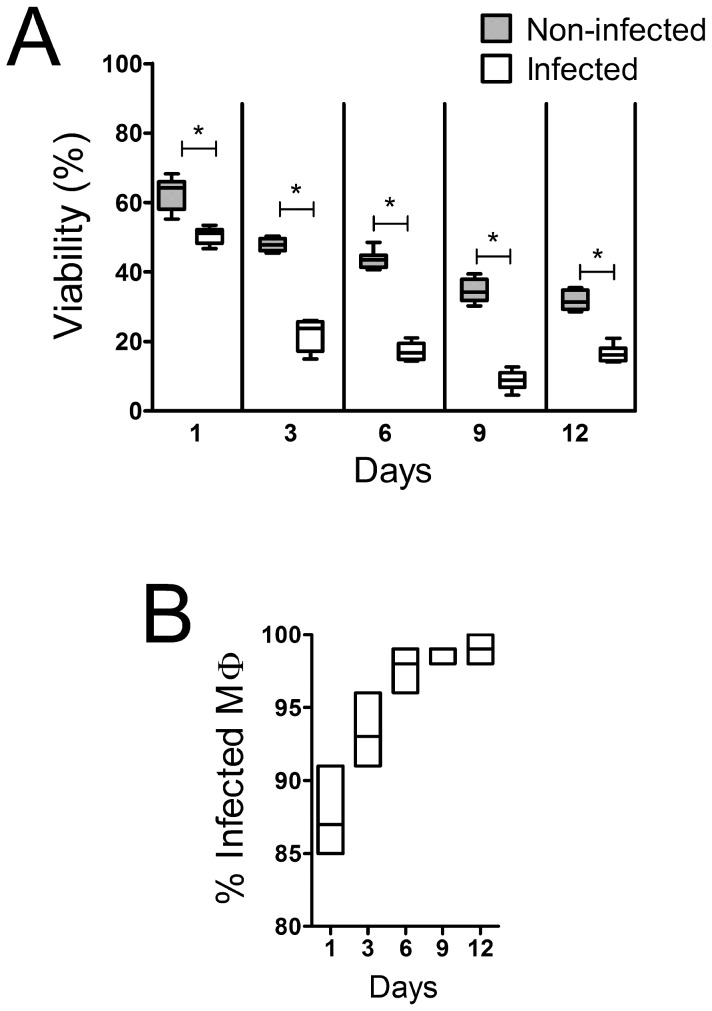
Macrophage viability and infection rate by *L. amazonensis*. A 50 parasites per macrophage ratio was used during the infection. A, percentage of chemical reduction of Alamar blue, relative to control, in macrophages infected with *L. amazonensis*. B, kinetics of infection. To determine the rate of infection, the macrophages were grown on coverslips, stained with HE, and the amastigotes visualized by optical microscopy (at least 200 macrophages were analysed per coverslip). The horizontal lines represent the median values of hexaplicates (A) or triplicates (B), the boxes the interquartile intervals (A) or the range (B), and the vertical bars the ranges (A). * p<0.05, Mann-Whitney test.

The infection of macrophages by *Leishmania* was evaluated by optical microscopy of H&E stained slides. The number of intracellular parasites increased with time. After nine days of culture, at the moment that the EVs were collected, about 100% of the cells were infected (Fig. 2A), with an average of 16 parasites per macrophage (not shown).

### Characterization of EVs and Annexin V-Staining of Macrophages

The EVs had diameters smaller than 1 µm, as demonstrated by flow cytometry in the presence of 1 µm-diameter beads (Fig. 1B). Their presence was demonstrated, by flow cytometry, in the supernatant of macrophages that had been labelled with PKH26 (Fig. 1C). The EVs were also stained with CFDA (not shown).

The EVs derived from these macrophages, infected or not with *L. amazonensis* and incubated for a period of 9 days, stained with antibodies specific for murine monocytes (anti-CD11b and anti-F4/80), as well as for the antigen-presenting molecule MHC II (Fig. 1D, E, F). About 45% of the EVs stained with Annexin V (Fig. 1G). There were no statistically significant differences between EVLa and EVmedium in terms of binding to Annexin V.

About 23% of infected macrophages stained with Annexin V, whereas a smaller proportion (14%) of uninfected macrophages stained with that reagent (not shown). In accordance with this finding, there was a more prominent reduction of macrophage numbers in infected cultures than in uninfected cultures (Fig. 2B).

**Figure 3:**
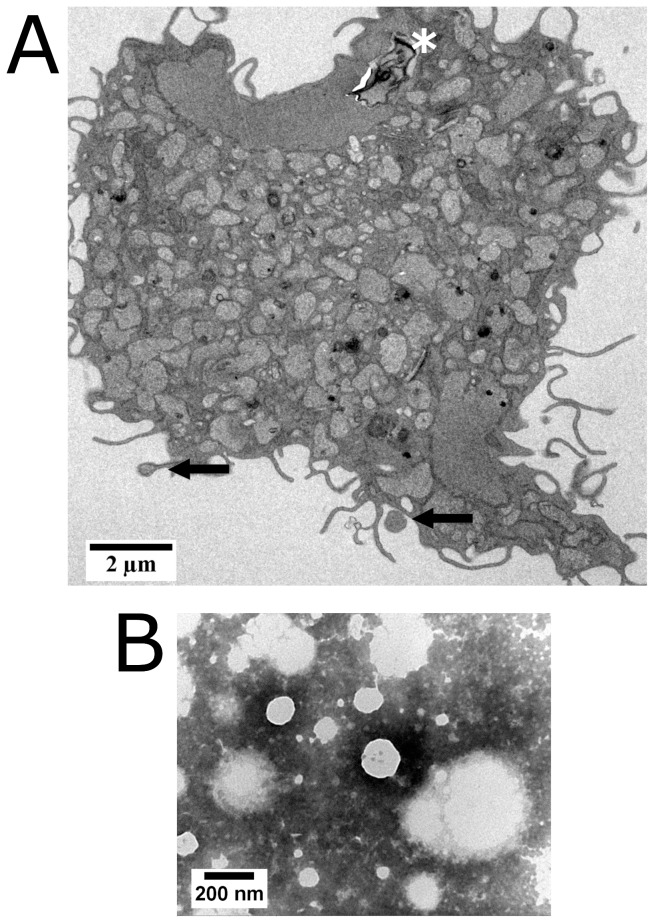
Transmission electron microscopy of extracellular vesicles (EVs) from supernatants of infected macrophage cultures and of *L. amazonensis*-infected macrophages presenting budding EVs. A, a macrophage was fixed with 2.5% glutaraldehyde and 2% paraformaldehyde and post-fixed with 1% osmium tetroxide, 0.8% potassium ferricyanide and 5 mM calcium chloride. The arrows indicate EVs being formed; the dark asterisk at the top of the figure indicates the position of the remainings of a *Leishmania* amastigote. Scale bar = 2 µm. B, EVs were adsorbed to copper grids (Formvar) for 20 minutes and negatively stained with 2% phosphotungstenic acid. Scale bar = 200 nm.

Transmission electron microscopy by negative contrast allowed the visualization of EVs and the observation of their heterogeneity in size. This visualization method has been described as appropriate for showing the presence of EVs [39]. The visualized EVs had spherical shapes and diameters ranging from 50 to 300 nm (Fig. 3A).

The macrophages infected with *L. amazonensis* were also evaluated by transmission electron microscopy, through which it was possible to visualize the formation of microparticles by blistering of the infected cell after nine days of culture (Fig. 3B).

The content of the vesicles had similar density to the cytosol and they were surrounded by cytoplasmic membrane (Fig. 3B).

### *Leishmania*-Infected and Uninfected Macrophages Release Comparable Amounts of EVs

The number of EVs was measured in the supernatants of infected and uninfected macrophages by flow cytometry in four independent experiments. The values acquired in the supernatants of 9-day cultures of 2×10^5^ infected macrophages (median = 5.0×10^7^; range = 3.5×10^7^ to 6.0×10^7^) and of the same number of uninfected macrophages (median = 5.2×10^7^; range = 4.7×10^7^ to 5.8×10^7^) did not differ significantly (p>0.05, Mann-Whitney test).

### EVs Derived from *L. amazonensis*-Infected Macrophages Induce the Production of Proinflammatory Cytokines by Naïve Macrophages

The ability of EVs derived from macrophages, infected or not with *L. amazonensis* for 9 days, to stimulate cytokine production by resident peritoneal macrophages was evaluated. The peritoneal macrophages were incubated with the EVs in the presence or absence of LPS for 48 hours. Cultures to which LPS was not added also contained polymyxin B in order to rule out the effect of endotoxin that could be contaminating the preparations. Similar results were observed in experiments with and without polymyxin B (data not shown).

**Figure 4:**
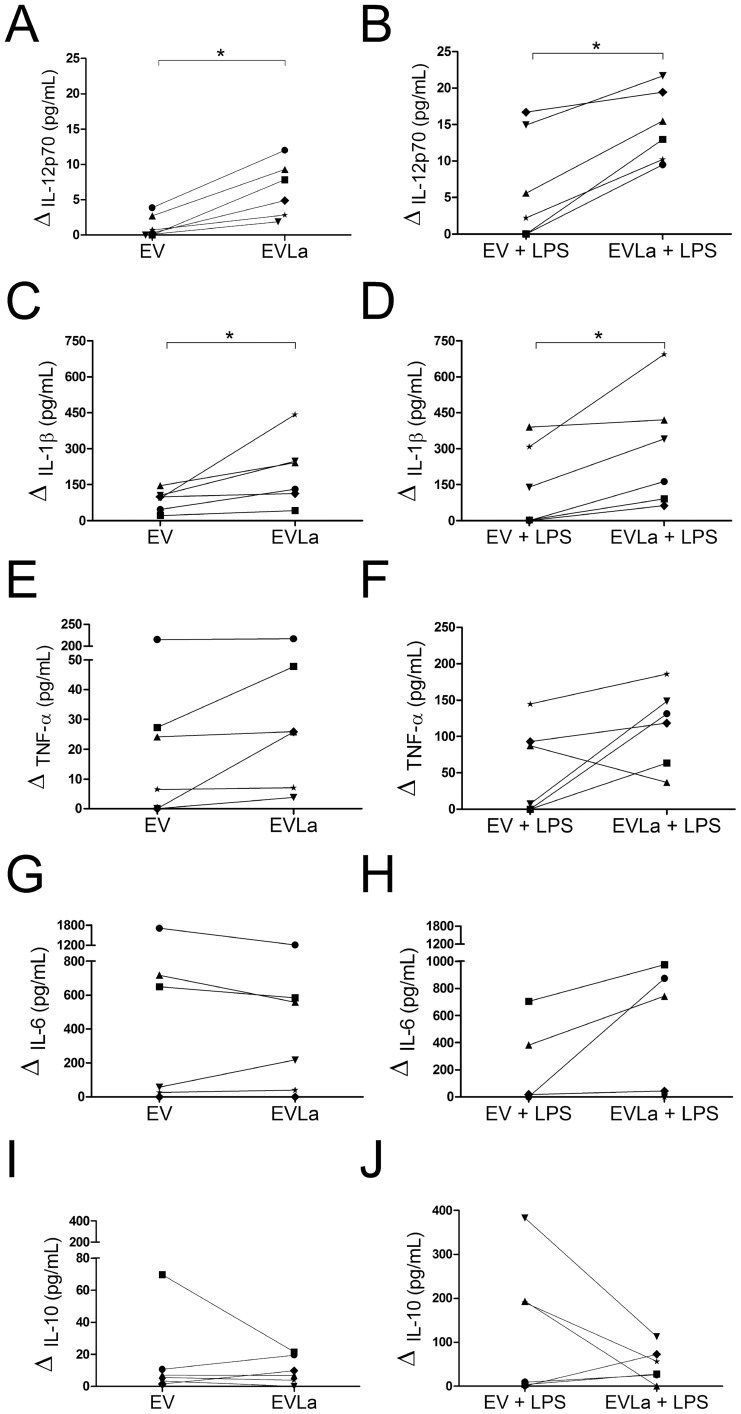
Effect of membrane vesicles from macrophages infected with *L. amazonensis* (EVLa) or not (EV) and stimulated with LPS or not on cytokine production by naïve macrophages. A and B, IL-12p70. C and D, IL-1β. E and F, TNF-α. G and H, IL-6. I and J, IL-10. A, C, E, G, and I, unstimulated macrophages; B, D, F, H, and J, macrophages stimulated with bacterium lipopolysacharide for 48 hours. The symbols and lines represent the averages of replicates of individual experiment. Data from six independent experiments are represented. Values obtained from cultures to which no EVs were added were subtracted from the data shown. *, P<0.025; Wilcoxon's signed-rank sum test.

Increased levels of IL-12p70 (i.e., levels of IL-12p70 above zero in the graphs) could be observed in cultures of EVLa-treated, unstimulated or LPS-stimulated macrophages in all six experiments (Figs. 4A and 4B). These increases (in relation to cultures to which no EVs were added) were statistically significant (p<0,025, Wilcoxon's signed-rank sum test). There was no statistically significant differences (p>0.05, Wilcoxon's signed-rank sum test) between the results obtained in the groups of cultures that received EVmedium in the six experiments, with and without LPS, and the results of their respective control groups (that received no EVs), despite the fact that increases in IL-12p70 levels could be observed in 2 out of 6 experiments in cultures of EVmedium-treated macrophages to which no LPS was added (Fig. 4A) and in 3 out of 6 experiments in LPS-stimulated cultures of EVmedium-treated macrophages (Fig. 4B).

The addition of both EVmedium and of EVLa to cultures without LPS led to the increased production of IL-1β in all six experiments (Fig. 4C; p<0.025, Wilcoxon's signed-rank sum test). On the other hand, the addition of EVmedium to LPS-stimulated cultures increased the production of IL-1β in only three out of six experiments (Fig. 4D, p>0.05, Wilcoxon's signed-rank test), whereas the addition of EVLa increased the production of IL-1β in all six experiments (Fig. 4D; p<0.025, Wilcoxon's signed-rank sum test).

When the cultures of macrophages treated with EVLa were compared with those treated with EVmedium in terms of the levels of IL-12p70 and IL-1β that were present in their supernatants in all six experiments, the addition of EVLa to both unstimulated and LPS-stimulated cultures led to higher levels of IL-12p70 and IL-1β than the addition of EVmedium (Figs. 4A, 4B, 4C, and 4D; p<0.025, Wilcoxon's signed-rank sum test). Notwithstanding this statistically significant difference, the difference in IL-1β levels in cultures of macrophages not stimulated with LPS when they were treated with EVLa or treated with EVmedium was minimal in 3 out of 6 experiments (Fig. 4C).

TNF-α levels were increased in cultures of EVLa-treated macrophages, treated or non-treated with LPS, in all six experiments (Figs. 4E and 4F; p<0.05, Wilcoxon's signed-rank sum test). TNF-α levels were also higher in cultures of EVmedium-treated macrophages, treated or non-treated with LPS, than in cultures to which no EVs were added, in 4 out of 6 and in 3 out of 6 experiments, respectively (Figs. 4E and 4F). However, these increases were not statistically significant (p>0.05, Wilcoxon's signed-rank sum test).

Clearly higher levels of TNF-α in cultures of EVLa-treated macrophages than in cultures of EVmedium-treated macrophages were observed in only two experiments in the absence of LPS (Fig. 4E) and in 5 out 6 experiments in the presence of LPS (Fig. 4F; p<0.05, Wilcoxon's signed-rank test).

No statistically significant differences in the levels of IL-6 and IL-10 in cultures to which EVmedium or EVLa were added were seen (Figs. 4G, 4H, 4I and 4J). Despite that, it can be noted that in 3 out of 6 cultures containing LPS, the concentrations of IL-10, an immune regulatory cytokine [40], [41], were lower with the addition of EVLa than when EVmedium was added (Fig. 4J). In the case of IL-6, a proinflammatory cytokine [42], the contrary was seen: in 3 out of 6 cultures containing LPS, the concentrations of that cytokine were markedly higher with the addition of EVLa than when EVmedium was added (Fig. 4H).

## Discussion

EVs have different membrane compositions, depending on the cell from which they have originated (reviewed in [10]). The fact that the EVs present in the supernatants of macrophages in this work had the surface markers CD11b and F4/80, as well as MHC class II, indicates their macrophage origin.

At least part of the EVs seems to have originated from apoptotic cells, since about 45% of both EVLa and EVmedium stained with Annexin V, which binds to phosphatidylserine molecules that are exposed in cell membranes during the apoptotic process [43]. The observation that some of the EVs were stained by Annexin V is consonant with the finding that a proportion of the macrophages were also stained by that reagent.

Staining by PKH26 confirmed the presence of lipid membranes associated with the EVs. The integrity of the EVs was demonstrated by flow cytometry with CFDA, a substance that permeates intact cell membranes and enters the cytosol, where it is converted by esterases into a green fluorescent derivative [44].

The formation of microparticles by blistering of macrophages infected with *L. amazonensis* could also be visualized by transmission electron microscopy. A similar formation of EVs in dendritic cells treated with LPS after 6 hours of cultivation was described by Obregon and collaborators [45].

The presence of *bona fide* EVs in the 100,000 g pellet of macrophage supernatants used in the present work was therefore confirmed by several parameters. Judging by their size (50 to 300 nm as shown by negative-staining transmission electron microscopy), the EVs could consist exclusively of microparticles or of microparticles and exosomes. Apoptoptic bodies could be ruled out, despite the staining of a proportion of the EVs with Annexin V, because they have diameters larger than 1 µm. As was mentioned in the Introduction, microparticles are also released by apoptotic cells. As around 45% of the EVs studied in this work stained with Annexin V, one can conclude that at least that amount of EVs are microparticles.

An interesting finding in the present work was that EVLa stimulated the production of IL-12p70, IL-1β, and TNF-α by uninfected macrophages. These macrophages could have been previously treated or not with LPS. Similar results were observed in experiments with macrophages not treated with LPS in the presence or in the absence of polymyxin B, indicating that putatively contaminating endotoxin did not participate in the obtained results. The carrying out of some experiments in the presence of bacterial LPS, which activates macrophages by leading to signal transduction by TLR4, was done in order to better emulate the *in vivo* situation, in which neutrophil elastase may stimulate macrophages via TLR4 [46].

The EVLa used in the present work were obtained from macrophages that were infected with a 50∶1 *Leishmania*∶macrophage ratio and cultured for 9 days *in vitro*. The possibility that the EVs released by macrophages infected with different numbers of parasites, or collected at different times after infection, differ in terms of biological activity from the EVs studied in the present work, should justify the carrying out of additional investigation.

A certain degree of variation in the pattern of secreted cytokines was observed among the six independent experiments that were carried out, both with the preparations of EVmedium and EVLa. This fact, which could depend on the batch of the EVs, on the responsiveness of the naïve macrophages, or on both, could have hindered the obtaining of statistically significant results for some cytokines. A great variation in the response of individual macrophage preparations to LPS was observed (data not shown), indicating that the responsiveness of the naïve macrophage preparation was, at least in part, responsible by the observed variations in the response to EVs. This variation did not, however, affect the acquisition of consistent results for IL-12p70, IL-1β, and TNF-α when the EVLa were tested.

One of the mechanisms of resistance of *L. amazonensis* to the immune system is an inhibition of IL-12 production by infected macrophages [47]. The effect of EVLa seen in this work suggests that a mechanism, namely the release of EVs might, at least in part, counteract this effect by inducing the production of IL-12, IL-1β and TNF-α by bystander macrophages before they are infected.

Thus, the contact of EVs from infected macrophages with non-infected macrophages, perhaps entailing the incorporation of these EVs, together with the possible carrying over of *Leishmania* antigens by the EVs to the naïve macrophages, could promote the differentiation of Th0 to Th1 lymphocytes, which would release IFN-γ, resulting in the Th1 response that is associated with resistance to the *Leishmania* infection [5], [48]. In addition, an increased production of IL-1β and TNF-α, which would also be induced in naïve macrophages by the EVs, is associated with host's resistance to *Leishmania* infection through the production of nitric oxide [49], [50]. Consistent with this, it has been reported that the injection of anti-TNF-α into mice worsened the *Leishmania* infection [51]. The phenomena described above could perhaps underlie the protective response that normally keeps the *Leishmania* parasites in check in most infected human beings [52], [53].

EVLa, therefore, are proinflammatory to macrophages. Macrophages infected with *Mycobacterium bovis* (BCG) release exosomes with capacity to generate proinflammatory immune responses *in vitro* and *in vivo* and that contain mycobacterial proteins [12], [27]. It could well be, therefore, that the proinflammatory nature could be a general feature of EVs originated from infected cells.

As mentioned above, the extracellular vesicle populations studied in the present work had sizes compatible with their being formed by a mixed population of microparticles and exosomes. It was recently reported that purified exosomes from both naïve and *Leishmania mexicana*-infected macrophages induce the activation of the pro-inflammatory transcription factors NF-kB and AP-1 [54]. Those infected macrophage-derived exosomes, however, induced slightly less activation of NF-kB than the naïve macrophage-derived exosomes. This apparent discrepancy with the present results could result from differences in the time after infection that the EVs were obtained, or from the fact that in the present work the total population of EVs was assayed, rather than only purified exosomes.

One possibility that was not ruled out in the present work is that the biological activity of the EVLa would be mediated by *Leishmania*-derived EVs, which has been described in the literature [29], [55]. However, contrary to the present observations, these *Leishmania* EVs had a suppressive effect on antigen-presenting cells [29], [55].

Since *Leishmania* parasites readily sediment at 1.500 g (unpublished observation), the centrifugation of the EVLa-containing supernatants at 8.000 g during the process of EVLa preparation eliminated the contamination of the EVLa preparations with *Leishmania*.

The naïve macrophage arming process described herein could perhaps be more intense during a *Leishmania* infection, in which EVLa would be released, but it seemed also to occur, in a smaller extent, with EVs from uninfected macrophages in the absence of LPS, at least as far as IL-1β is concerned (Fig. 4C). Whether this apparently spontaneous release of EVs that stimulate the production of IL-1β is an *in vitro* artefact or a relevant phenomenon *in vivo* is open to speculation. EVs are certainly released in physiological contexts, as can be exemplified by the finding of circulating EVs in healthy human beings' plasma samples [56].

In the present work, in an attempt to perhaps emulate what could occur in a natural infection, the concentrations in which the effect of the EVs were assessed on naïve macrophages were the same ones that were present in the supernatants from the macrophages that released them (i.e. the EVs were not concentrated nor diluted). Similar amounts of EVLa and EVmedium were added to the cultures, showing that the higher induction of production of pro-inflammatory cytokines by EVLa than by EVmedium is due to qualitative differences between the two kinds of EVs.

One of the possible mechanisms that could account for the observed effect of the EVs would be their ligation to receptor of danger-associated molecular patterns (DAMPs) on the macrophage surface. EVs could present DAMPs from apoptotic or necrotic cells, such as lectin C, which is expressed in dead cells or in cell in death process [57]. As presented above, there is evidence that apoptosis might be involved in the generation of at least part of the EVLa. It has been reported that apoptotic bodies induce immune responses (reviewed in [36]). Other components that could be carried over by the EVS, such as uric acid and cholesterol crystals, are recognized by NLRP3 receptors [58], activating the NLRP3 inflammasome and leading to the production of proinflammatory cytokines. The activation of that inflammasome has been shown to lead to the production of IL-1β e IL-1α [59]. Another possibility that is open to investigation is that *Leishmania* molecules, possibly carried out by the EVs, could be triggering the pro-inflammatory cytokine production.

It has been reported in the literature that the *in vitro* infection by Leishmania increased the resistance of macrophages to the development of apoptosis [60], [61]. These reports are in apparent contradiction with the present findings that a larger proportion of infected macrophages had exposed phosphatidylserine on their cell membranes (i.e., they stained with Annexin V), a fact that is an indication of apoptosis, than uninfected macrophages. However, the cited reports studied macrophages that have been infected for no more than 24 hours, whereas in the present work the macrophages were infected for 9 days, in an attempt to emulate what probably occurs *in vivo*, when heavily infected macrophages are found and the *Leishmania* lesions usually last for months. It is possible that, early in the infection, the *Leishmania* may induce anti-apoptotic mechanisms that are important to allow the host cell to survive long enough to allow the parasite to multiply and to spread to other cells and to the insect vector. However, late in the infection, when the host cells are heavily loaded with parasites, this anti-apoptotic activity would perhaps no longer prevail — anyway, it would no longer be relevant for the parasite life cycle, and maybe would even hinder its continuity.

One possible exploitation of proinflammatory EVs is their use in vaccination and immunotherapeutic procedures, since they may carry MHC-antigenic peptide complexes, unbound antigens [10], cytokines that intensify the immune response and/or change its nature, costimulatory molecules [13], [19], [27], and, as shown in the present work, a still unidentified factor that induces the formation of proinflammatory cytokines, including IL-12, which may serve as an adjuvant for Th1 immune responses.

The ability of infected macrophage-derived proinflammatory EVs in conferring a resistant phenotype to *Leishmania* infection *in vitro* and *in vivo*, and the possible presence of *Leishmania* antigens in them, are currently under investigation.

### Conclusions

As shown in the present work, EVs from macrophages infected with *L. amazonensis* induce the production of proinflammatory cytokines by naïve macrophages, and may, therefore, play a role in stimulating the immune response and in conferring a resistant phenotype to naïve, bystander macrophages.
