# Correction: Extracellular Vesicles from *Leishmania*-Infected Macrophages Confer an Anti-infection Cytokine-Production Profile to Naïve Macrophages

**DOI:** 10.1371/journal.pntd.0003371

**Published:** 2014-11-05

**Authors:** The PLOS Neglected Tropical Diseases Staff

The sixth author’s name is spelled incorrectly. The correct name is: Cláudio P. Figueira.
